# Diabetes Screening in US Women With a History of Gestational Diabetes, National Health and Nutrition Examination Survey, 2007–2012

**DOI:** 10.5888/pcd13.160106

**Published:** 2016-09-08

**Authors:** Bernice Man, Mary E. Turyk, Michelle A. Kominiarek, Yinglin Xia, Ben S. Gerber

**Affiliations:** Author Affiliations: Mary E. Turyk, School of Public Health, University of Illinois at Chicago, Chicago, Illinois; Michelle A. Kominiarek, Department of Obstetrics and Gynecology, Northwestern University Feinberg School of Medicine, Chicago, Illinois; Yinglin Xia, Ben S. Gerber, Department of Medicine, University of Illinois at Chicago, Chicago, Illinois;

## Abstract

**Introduction:**

Women with a history of gestational diabetes mellitus (GDM) are at increased risk for developing type 2 diabetes mellitus. We examined individual, socioeconomic, and health care use characteristics of women with a history of GDM and the association of those characteristics with diabetes screening, and we estimated their rates of undiagnosed prediabetes and diabetes.

**Methods:**

Using 3 cycles of the National Health and Nutrition Examination Survey (2007–2008, 2009–2010, and 2011–2012), we identified 284 women with a history of GDM who were eligible for diabetes screening. Screening status was defined by self-report of having had a blood test for diabetes within the prior 3 years. Undiagnosed prediabetes and diabetes were assessed by hemoglobin A_1c_ measurement.

**Results:**

Among women with a history of GDM, 67% reported diabetes screening within the prior 3 years. Weighted bivariate analyses showed screened women differed from unscreened women in measured body mass index (BMI) category (*P* = .01) and number of health visits in the prior year (*P* = .001). In multivariable analysis, screening was associated with a greater number of health visits in the prior year (1 visit vs 0 visits, adjusted odds ratio [AOR], 1.91; 95% confidence interval [CI], 0.71–5.18; 2 or 3 visits, AOR, 7.05; and ≥4 visits, AOR, 5.83). Overall, 24.4% (95% CI, 18.3%–31.7%) of women had undiagnosed prediabetes and 6.5% (95% CI, 3.7%–11.3%) had undiagnosed diabetes.

**Conclusion:**

More health visits in the prior year was associated with receiving diabetes screening. Fewer opportunities for screening may delay early detection, clinical management, and prevention of diabetes. Prediabetes in women with a history of GDM may be underrecognized and inadequately treated.

## Introduction

Gestational diabetes mellitus (GDM) is defined as the onset or first recognition of diabetes during pregnancy, typically diagnosed by an abnormal oral glucose tolerance test (OGTT) during the second trimester (1,2). Prevalence of GDM ranges from 2% to 10% (3–5). Although glucose intolerance resolves immediately after delivery in 90% of women with GDM, their risk of developing type 2 diabetes mellitus is 35% to 60% within 5 to 10 years (6,7), which is a 5- to 7-fold increase in risk compared with women without a history of GDM. The American Diabetes Association and American College of Obstetricians and Gynecologists recommend diabetes screening at 6 to 12 weeks postpartum with a 2-hour, 75-g OGTT (1,2). Moreover, both organizations recommend lifelong screening for diabetes at least once every 3 years and annual screening for those with prediabetes (1,2).

Approximately 50% of women with a history of GDM obtain diabetes screening, with rates ranging from 30% to 70% (8). Screening with the recommended OGTT is uncommon (9); most studies recognize or consider any marker of glucose measure as a screening test. A study conducted at a university health care system found a screening rate of 38% with any test of glucose marker at least 6 weeks postpartum during a 5-year period (10). Another study found 67% of women with previous GDM had some type of screening, but only 37% were tested with an OGTT or fasting blood glucose within 2 years (11).

Prior studies found that postpartum diabetes screening among US women was positively associated with women who were Asian, had high levels of education or income, were diagnosed with GDM at a young age, and had a high number of health care provider contacts (12,13). However, higher parity was associated with lower screening rates (12,13). Findings from previous US studies have been limited to single academic centers or managed care organizations subject to local practice patterns and policies. Also, little is known about the glycemic status and characteristics of women with GDM who disengage from the health care system after delivery (14).

Our study objectives were to use a nationally representative data set 1) to determine the diabetes screening rate and identify characteristics associated with screening and 2) to determine the proportion of women with a history of GDM with undiagnosed prediabetes and diabetes.

## Methods

### Study design and oversampling

The National Health and Nutrition Examination Survey (NHANES) is an ongoing cross-sectional survey of the civilian, noninstitutionalized US population. Participants are selected through a complex multistage probability cluster sampling design. Sampling methodology and data collection procedures have been published in detail (15). Publicly released data from 3 NHANES cycles — 2007–2008, 2009–2010, and 2011–2012 — were combined for analysis. Beginning in 2007, Hispanics were oversampled, and beginning in 2011, Asians were oversampled to improve the reliability and precision of estimates for these population subgroups. Thus, estimates of GDM prevalence among Asians were limited to the 2011–2012 cycle, and for the full analysis, race/ethnicity was categorized as non-Hispanic white, non-Hispanic black, Hispanic, and other/multiracial.

### Survey components

Survey components included interviews administered at home for all participants and a visit to the mobile examination center (MEC) for a subsample. A standardized physical examination, laboratory tests, and the administration of the reproductive health questionnaire to female participants aged 12 years or older via computer-assisted personal interview were conducted in the MEC. Data from those aged 12 to 19 years for select reproductive health variables were excluded from public files because of disclosure concerns. Hemoglobin A1c (HbA_1c_) level was measured, and body mass index was calculated based on measured height and weight for each MEC participant. Fasting glucose and OGTT were measured in a subsample of the MEC participants but were not included in the analysis because of the small number of eligible women selected for the fasting protocol.

### Variable selection

We examined the following variables: race/ethnicity, age, education level, family income-to-poverty ratio (FIPR), marital status, BMI, foreign-born status, language preference, age at GDM diagnosis, health insurance status, type of health insurance, place used for routine health care, number of health care visits in prior year, number of pregnancies, number of live births, age at first and last birth, and having a baby with a birthweight of 9 pounds or greater. A non-English language preference was defined for participants who reported a non-English language spoken at least 50% of the time at home or if a non-English language was used for any part of the survey. Education level and FIPR were examined as measures of socioeconomic status in the bivariate analysis. The FIPR is calculated by dividing annual family income by poverty guidelines specific to family size, year, and state, and is recommended for comparing income data over time (15). In our analysis, the FIPR was categorized by the Special Supplemental Nutrition Program for Women, Infants and Children (WIC) program eligibility criteria (15). In multivariable analyses, education level was categorized as either high school graduate or less than high school graduate.

### Cohort selection for women with a history of GDM

A total of 30,442 participants were enrolled in NHANES 2007–2012. Our cohort was selected from the 29,353 individuals who attended the MEC portion of the survey (mean MEC response rate for the 3 cycles was 74%). After excluding males and females less than 20 years old, a total of 8,739 women were eligible for the reproductive survey. Of those eligible, 6,516 reported having been pregnant or having had at least 1 pregnancy; 1,075 had missing responses. Nonresponders included those who did not report to the MEC or did not provide an answer to the question “Have you ever been pregnant?” Women who reported at least 1 prior pregnancy were asked “Were you ever told by a doctor or other health professional that you had diabetes, sugar diabetes or gestational diabetes? Please do not include diabetes that you may have known about before the pregnancy.” Excluded from the analysis were women who answered no (n = 5,996), “don’t know” (n = 12), and “borderline” (n = 75). Women who had a positive pregnancy test at the MEC or reported a current pregnancy were excluded (n = 12). There were 421 women who reported a history of GDM (Figure, group A).

**Figure:**
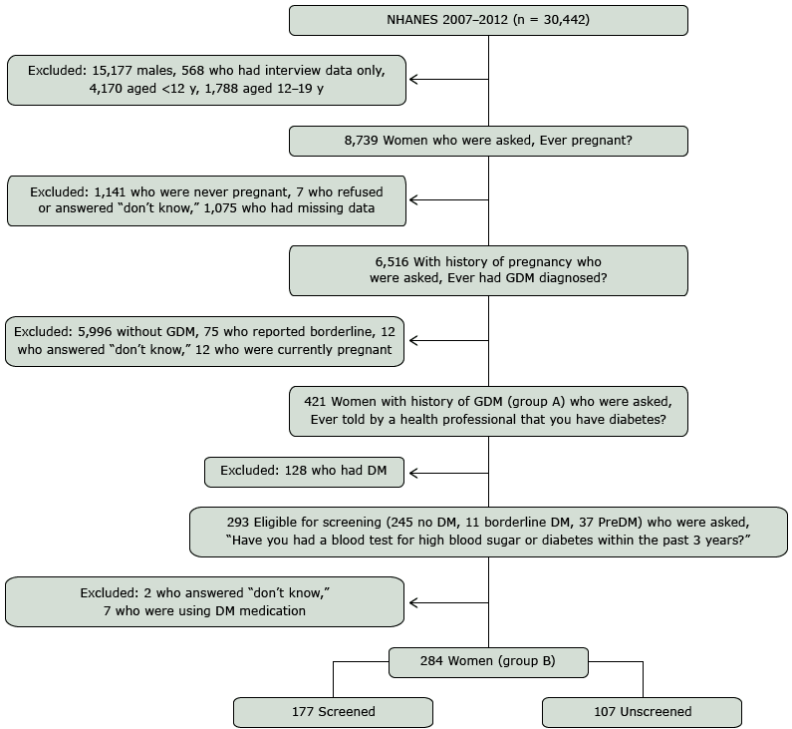
Flow diagram for cohort selection of women with GDM screened or unscreened for DM, NHANES, 2007–2008, 2009–2010, and 2011–2012. Abbreviations: DM diabetes mellitus; GDM, gestational diabetes mellitus; NHANES, National Health and Nutrition Examination Survey; PreDM, prediabetes.

Women with a history of GDM were considered eligible for diabetes screening if they did not have a self-reported diagnosis of diabetes. Of the 421 women with a history of GDM, 128 women reported a diagnosis of diabetes and were excluded from the analytic subsample. Women who reported a diagnosis of borderline diabetes or prediabetes (n = 48) were included; however, those who reported using medications to lower blood glucose (n = 7) were excluded from the analytic subsample. Diabetes screening status was determined by the response to “Have you had a blood test for high blood sugar or diabetes within the past 3 years?” Women who responded “don’t know” to prior screening (n = 2) were excluded from our analytic subsample (n = 284, Figure, group B). Of the 284 nondiabetic, at-risk women eligible for diabetes screening, 177 reported having had a blood test for diabetes within the prior 3 years.

Women who reported no prior diagnosis of prediabetes or borderline diabetes but whose HbA_1c_ was 5.7% to 6.4% (39–46 mmol/mol) were characterized as having undiagnosed prediabetes. Women who had an HbA_1c_ of 6.5% or higher (≥47.5 mmol/mol) were characterized as having undiagnosed diabetes.

### Statistical analysis

All statistical analyses used survey design variables and were weighted with the examination subsample MEC 6-year weight to account for the complex sampling scheme, oversampling, and survey nonresponse to produce nationally representative estimates per NHANES analytic guidelines (15). We generated age-adjusted prevalence and standard errors for women with a history of GDM (Figure, group A) with the direct method for age standardization using 2000 US Census population data for those aged 20 years or older, with 10-year age intervals (15). Next, we performed bivariate analyses of cohort characteristics with diabetes screening. Associations of categorical variables were analyzed by χ^2^ test of independence. Associations of normally distributed continuous variables — survey age and age at first pregnancy — were assessed by *t* test. For nonnormally distributed continuous variables — age at GDM diagnosis and age at last birth — a nonparametric *t* test (Wilcoxon rank sum) was used to assess the association. All tests were 2-tailed, and *P* < .05 was considered significant.

We constructed multivariable logistic regression models to examine the association of diabetes screening with independent variables, adjusted for age, education, income, race/ethnicity, BMI category, and age at GDM diagnosis. A backward elimination approach was used to fit significant independent variables to achieve the most parsimonious model. Independent variables were evaluated for collinearity by examining the variance inflation factor before inclusion in the models. We used Stata version 13.1 (StataCorp LP) for all analyses. The institutional review board of the University of Illinois at Chicago reviewed the study protocol and determined this study exempt from human subjects research oversight.

## Results

### Prevalence of GDM in the US population

The estimated age-standardized prevalence of GDM was 7.3% (95% confidence interval [CI], 6.3%–8.2%) for US women aged 20 or older during 2007–2012. Thirty-six percent of the women were less than 40 years old at the time of the survey, and more than half (56%) were obese (BMI ≥30 kg/m^2^). Weighted and age-standardized (for 10-year intervals) prevalence of having had diabetes diagnosed was 24%. Age-standardized prevalence was highest among Hispanics (8.5%; 95% CI, 6.5%–10.6%), followed by non-Hispanic whites (6.9%; 95% CI, 5.4%–8.4%) and non-Hispanic blacks (6.6%, 95% CI, 5.4%–7.8%). Among racial/ethnic categories, GDM prevalence was highest among women categorized as other/multiracial (9.5%; 95% CI, 6.1%–12.8%). This high-prevalence group consisted of multiracial and other race/ethnicities, including Asians. Using only data collected from the 2011–2012 cycle in which Asian race was reported and Asians were oversampled, the age-standardized GDM prevalence among Asians was 11.1% (95% CI, 7.4%–14.8%).

### Rates of diabetes screening among women with a history of GDM

Sixty-seven percent (95% CI, 58.9%–75.1%) of women with a history of GDM without a diagnosis of diabetes reported blood test screening for diabetes within the prior 3 years (weighted and age-standardized prevalence). A greater number of visits to a health provider in the prior year was associated with diabetes screening among women with a history of GDM (*P* = .002) (Table 1). Screened women differed from unscreened women by BMI category: 53.6% versus 35.7%, respectively, were obese; 18.6% versus 41.6% were overweight; and 27.8% versus 22.8% were underweight or normal weight (*P* = .01).

**Table 1:** Weighted Bivariate Analysis of Characteristics of Women With a History of Gestational Diabetes Mellitus (n = 284), by Diabetes Screening Status, NHANES, 2007–2012^a^

Variable	Screened (n = 177)	Unscreened (n = 107)	*P* Value^b^
**Mean age at time of survey (SE), y**	42.4 (0.9)	42.3 (1.1)	.91^c^
**Age group, % (95% CI), y**			
20–39	45.6 (37.5–53.8)	44.5 (35.3–54.1)	.51
40–59	47.6 (38.2–57.1)	51.7 (41.9–61.4)	
≥60	6.9 (3.2–14.1)	3.8 (1.6–8.8)	
**Mean age at GDM diagnosis (SE), y**	28.1 (0.57)	27.3 (0.59)	.45^d^
**Not married,^e^ % (95% CI)**	23.0 (16.1–31.7)	25.1 (15.2–38.7)	.76
**Race/ethnicity, % (95% CI)**			
Non-Hispanic white	62.5 (51.8–72.2)	66.5 (53.2–77.5)	.08
Hispanic	14.6 (9.3–22.4)	21.1 (13.5–31.5)	
Non-Hispanic black	13.6 (10.2–17.9)	6.7 (3.4–13.1)	
Other/multiracial^f^	9.3 (5.0–16.6)	5.7 (2.5–12.5)	
**Not US born, % (95% CI)**	21.5 (13.9–31.8)	22.4 (13.9–34.0)	.88
**Preferred language, % (95% CI)**			
English	81.9 (72.7–88.5)	83.3 (74.1–89.7)	.07
Spanish	11.8 (6.9–19.4)	14.9 (8.9–24.0)	
Asian or other	6.3 (2.9–13.1)	1.8 (0.7–4.3)	
**Education level, % (95% CI)**			
Less than 9th grade	4.9 (2.2–10.5)	10.4 (5.9–17.7)	.39
9th to 11th grade	12.0 (7.2–19.2)	14.2 (8.4–23.2)	
High school graduate	22.0 (14.2–32.4)	25.1 (16.0–37.1)	
Some college	32.5 (24.8–41.4)	32.8 (22.8–44.7)	
College graduate or more	28.6 (18.8–40.9)	17.4 (9.2–30.7)	
**High school graduate or more, % (95% CI)**	83.1 (74.5–89.2)	75.4 (66.3–82.7)	.15
**Family income-to-poverty ratio,^g^ % (95% CI)**			
0–1.85	31.4 (23.9–40.1)	42.7 (29.7–56.8)	.21
>1.85–3.5	22.6 (15.7–31.4)	26.9 (15.2–43.0)	
≥3.5	46.0 (36.0–56.2)	30.4 (18.8–45.3)	
**Health insurance, % (95% CI)**	80.5 (72.3–86.7)	71.3 (61.4–79.5)	.13
**Insurance coverage type, % (95% CI)**			
Private	63.7 (54.5–72.1)	57.4 (45.8–68.2)	.31
Medicaid	7.1 (4.7–10.5)	4.7 (2.3–9.2)	
Medicare/Medigap	2.5 (1.1–5.6)	4.0 (1.7–9.2)	
Other	7.2 (3.7–13.5)	5.3 (2.5–10.8)	
No coverage	19.5 (13.3–27.8)	28.7 (20.6–38.6)	
**Have a place for routine health care, % (95% CI)**	88.3 (80.5–93.3)	78.5 (66.4–87.1)	.12
**Place often used for routine health care, % (95% CI)**			
Clinic or health center	18.9 (13.4–25.9)	20.9 (13.5–30.8)	.24
Office or health maintenance organization	64.9 (56.3–72.6)	55.3 (42.7–67.2)	
Other (emergency department, hospital, urgent care)	4.6 (1.9–10.6)	2.4 (0.79–7.1)	
None	11.7 (6.7–19.5)	21.5 (12.9–33.6)	
**No. of health care visits in prior year, % (95% CI)**			
None	9.3 (5.2–16.1)	24.2 (14.9–36.8)	.002
1	14.6 (8.8–23.3)	30.7 (19.5–44.8)	
2 or 3	36.5 (29.0–44.7)	20.5 (12.2–32.4)	
≥4	39.6 (31.7–48.1)	24.6 (17.0–34.2)	
**No. of pregnancies, % (95% CI)**			
1	12.8 (7.1–22.0)	10.6 (4.6–22.5)	.93
2	25.2 (16.9–35.9)	25.5 (15.7–38.6)	
3	26.4 (18.9–35.5)	24.5 (16.9–34.3)	
>4	35.6 (27.8–44.3)	39.3 (29.1–50.6)	
**No. of live births, % (95% CI)**			
0	0.3 (0.0–2.2)	0	.51
1	24.3 (15.9–35.3)	16.7 (8.2–31.0)	
2	39.6 (28.4–52.0)	38.9 (27.8–51.1)	
3	22.4 (16.3–30.0)	30.5 (27.8–51.1)	
≥4	13.4 (9.0–19.5)	14.0 (8.5–22.0)	
**Mean age at first birth (SE), y**	23.1 (0.6)	23.2 (0.6)	.91^c^
**Mean age at last birth, y (SE)**	30.4 (0.5)	30.1 (0.6)	.74^d^
**Had baby with birthweight ≥9 pounds, % (95% CI)**	29.9 (20.4–41.4)	30.6 (18.8–45.7)	.93
**BMI (kg/m^2^), % (95% CI)**			
<25.0 (Underweight or normal weight)	27.8 (20.5–36.4)	22.8 (13.6–35.6)	.01
25.0–29.9 (Overweight)	18.6 (11.9–27.9)	41.6 (30.6–53.4)	
≥30.0 (Obese)	53.6 (44.2–62.8)	35.7 (25.7–47.1)	
**HbA_1c,_ % (95% CI)**			
<5.7 (<39 mmol/mol)	68.6 (59.2–76.8)	67.7 (56.1–77.4)	.96
5.7–6.4 (39–46 mmol/mol)	24.2 (17.0–33.1)	25.8 (16.2–38.5)	
≥6.5 (≥47.5 mmol/mol) (Undiagnosed diabetes)	7.2 (3.5–14.0)	6.5 (2.9–13.9)	
**Undiagnosed prediabetes, % (95% CI)**	22.4 (15.6–31.0)	25.8 (16.2–38.5)	.59

Abbreviations: BMI, body mass index; CI, confidence interval; GDM, gestational diabetes; HbA_1c_, hemoglobin A_1c_; NHANES, National Health and Nutrition Examination and Survey; SE, standard error.

a All percentages are weighted.

b χ^2^ test of independence unless otherwise noted.

c Unpaired 2-tailed *t* test; degrees of freedom = 49 for mean age at time of survey, 48 for mean age at first birth.

d Wilcoxon rank sum test.

e Single, separated, or divorced.

f Beginning in 2011, Asians were oversampled to improve the reliability and precision of estimates for these population subgroups.

g Based on eligibility categories for Special Supplemental Nutrition Program for Women, Infants and Children (15).

After adjusting for age, race/ethnicity, education, FIPR, BMI category, and age at GDM diagnosis, diabetes screening was associated with having a greater number of health provider visits in the prior year, having a higher income (FIPR >3.5), and preferring a language other than English or Spanish (Asian or other). The odds of screening were higher among women who reported 2 or 3 visits (adjusted odds ratio [AOR], 7.05; 95% CI, 2.18–22.8) and those reporting 4 or more visits (AOR, 5.83; 95% CI, 2.35–14.46) compared with women who reported 0 visits (Table 2).

**Table 2:** Multivariable Logistic Regression Model Examining the Association Between Diabetes Screening and Selected Independent Variables, NHANES, 2007–2012

Independent Variable	AOR (95% CI)	*P* Value
**Age at time of survey **	0.98 (0.95–1.01)	.12
**Age at GDM diagnosis**	1.04 (0.99–1.10)	.10
**Race/ethnicity**		
Non-Hispanic white	1 [Reference]	
Hispanic	0.50 (0.13–1.86)	.29
Non-Hispanic black	1.93 (0.81–4.62)	.14
Other/multiracial^a^	0.61 (0.17–2.18)	.44
**High school graduate**	1.17 (0.61–2.24)	.63
**Family income-to-poverty ratio^b^**		
0–1.85	1 [Reference]	
>1.85–3.5	1.46 (0.58–3.57)	.43
>3.5	2.49 (1.07–5.78)	.03
**BMI (kg/m^2^)**		
<25.0	1 [Reference]	
25.0–29.9	0.58 (0.22–1.53)	.26
≥30.0	1.63 (0.69–3.85)	.26
**Preferred language**		
English	1 [Reference]	
Spanish	3.34 (0.66–16.85)	.14
Asian or other	8.93 (1.55–51.44)	.02
**No. of health care visits in prior year**		
None	1 [Reference]	
1	1.91 (0.71–5.18)	.20
2 or 3	7.05 (2.18–22.8)	.002
≥4	5.83 (2.35–14.46)	<.001

Abbreviations: AOR, adjusted odds ratio; BMI, body mass index; CI, confidential interval; GDM, gestational diabetes mellitus; NHANES, National Health and Nutrition Examination and Survey.

a Beginning in 2011, Asians were oversampled to improve the reliability and precision of estimates for these population subgroups.

b Based on eligibility categories for Special Supplemental Nutrition Program for Women, Infants and Children (15).

### Rates of undiagnosed prediabetes and undiagnosed diabetes

Fourteen percent (weighted percentage) of women with prior GDM (Figure, group A) reported a diagnosis of prediabetes or borderline diabetes. Of the 62% of women who reported normal glycemic status (no diabetes, prediabetes, or borderline diabetes), 24.4% (95% CI, 18.3%–31.7%) had undiagnosed prediabetes and 6.5% (95% CI, 3.7%–11.3%) had undiagnosed diabetes. Overall, one-third of the women with a history of GDM had diagnosed or undiagnosed prediabetes. We found no differences in the proportion of undiagnosed prediabetes or undiagnosed diabetes between the screened and unscreened groups (Table 1).

## Discussion

A greater number of health visits in the prior year was associated with diabetes screening after adjustment for other characteristics such as age, race/ethnicity, education, income, BMI category, and age at GDM diagnosis. Our findings suggest unscreened women may disengage from the health care system after delivery. Prior qualitative studies reported a lack of time and childcare as perceived practical barriers to screening, but some women also expressed fear of a diagnosis of diabetes or were uninformed of their risk (16–18). Moreover, the recommended interval for cervical cancer screening has lengthened to every 3 to 5 years, such that postpartum women age 30 years or older may seek preventive health care less frequently (19). Inadequate or lack of health care coverage may cause some women to involuntarily disengage from the health care system. Approximately 50% of pregnancies are covered by Medicaid (14,20). Before Medicaid’s expansion under the Patient Protection and Affordable Care Act, health visits more than 60 days after pregnancy for diabetes screening and care were not available to women in some states, which may explain the low rate of postpartum screening observed among women with Medicaid (14). However, we did not find an association between type of health insurance and screening status in our analysis.

Evidence also suggests that screening is not performed even when women are engaged with the health care system (9,11), that is, disengagement is unlikely to be a barrier to screening. Smirnakis and colleagues showed that more than 94% of women had a Papanicolaou test, but only 37% underwent postpartum diabetes screening within 6 months (11). Provider unawareness of GDM diagnosis, fragmentation in care, and nonadherence to guidelines may contribute to suboptimal screening rates (10,12,21,22). The implementation of electronic clinical support did not increase the rate of diabetes screening (23). Only one-third of women with GDM were referred for a screening test or given a referral to a primary physician by their obstetrician/gynecologist (24). Our findings also suggest that more contact with the health care system presents more opportunities for screening or that screening may occur in conjunction with other provider-directed evaluations. Among those screened, 39.6% had 4 or more health care visits in the prior year. The rising prevalence of diabetes and obesity has contributed to a greater awareness of these conditions among the general public and may have prompted health providers to screen for diabetes more often, irrespective of a GDM diagnosis. Providers consider obesity a key risk factor for diabetes, and obese women are more frequent users of health care services (25). We found higher rates of obesity among screened women than among unscreened women (53% vs 35.7%). However, one-fourth of the women in the NHANES were underweight or normal weight, underscoring the reality that some women may not have an obvious diabetes risk factor.

Competing issues that may steer the agenda of the health care visit toward more urgent concerns and away from preventive screening were not studied. More health encounters may offer exposure to different providers, to different specialists, or to more opportunities to address preventive care such as diabetes screening. Encouraging routine health care use and improving the transition from postpartum care to primary care may also contribute to better screening rates.

Higher income level was also associated with screening. Women who preferred speaking another language other than Spanish or English were also more likely to be screened. Presumably, this finding suggests that language may not be a uniform barrier to screening.

Approximately one-quarter of US women with a history of GDM had undiagnosed prediabetes, and approximately 6.5% had undiagnosed diabetes. None of these women were taking diabetes medications and none reported a prior diagnosis of diabetes, prediabetes, or borderline diabetes. Furthermore, the number of women with undiagnosed prediabetes is likely an underestimation because of the lower sensitivity of the HbA_1c_ test. Of the women with a history of GDM, 14% reported a diagnosis of prediabetes or borderline diabetes, but only 8.6% reported the use of glucose-lowering medications. In a subgroup analysis of the Diabetes Prevention Program, women with a history of GDM and with prediabetes were 48% more likely to progress to diabetes compared with women with similar glucose intolerance without GDM (26). In women with a history of GDM, intensive lifestyle intervention and metformin reduced progression to diabetes by 35% and 40%, respectively, compared with placebo (26). Metformin may be more effective in prediabetic women with a history of GDM compared with similar women without GDM (26,27).

These findings support the American Diabetes Association position on prediabetes screening and treatment (1). Our findings show the distribution of HbA_1c_ testing was not statistically different among screened and unscreened women. Prediabetes awareness in adults is associated with a greater likelihood of engaging in risk-reducing behaviors (28). However, the use of metformin in the prevention of diabetes is uncommon (29). Prediabetes in women with a history of GDM may be underrecognized and inadequately treated.

Early intervention offers reproductive-age women opportunities to optimize any glucose intolerance during their interconception period, potentially decreasing subsequent diabetes-related pregnancy complications. Delaying the onset of or preventing diabetes may have profound and prolonged effects in the health and productivity of these women in later life (30). Therefore, women of reproductive age with a history of GDM may need to be screened more frequently. An emphasis on prediabetes screening may be considered in this high-risk population given the high risk of progression to diabetes and positive response to intervention (26). Systematic methods to improve prediabetes screening are being investigated.

Our study had numerous strengths and limitations. The use of data from a large nationally representative sample allowed us to produce population estimates. Survey administration, laboratory measurements, and medical examinations were conducted by highly trained personnel using standardized protocols. Survey data was self-reported with the exception of data on weight, height, and HbA_1c_. The diagnosis of GDM, history of health care usage, and the performance of diabetes screening were not verified. Temporal changes in the diagnostic criteria of GDM and standard of care for diabetes screening in these women were not accounted for in the analysis; however, we noted no significant differences in screening by survey cycle. The ability to detect significant differences between screened and unscreened women may have been limited because of our small sample size. We were unable to examine screening characteristics of Asians and Asian language preference separately. However, using only data collected from the 2011–2012 cycle in which Asian race was reported and Asians were oversampled, the age-standardized GDM prevalence among Asians was 11.1% (95% CI, 7.4%–14.8%). This may contribute to the high GDM prevalence noted in our other/multiracial ethnic category.

Women with GDM reporting higher health care usage were more likely to report diabetes screening. Limited engagement with the health care system likely reduces opportunities for screening. An emphasis on increasing prediabetes screening may also delay progression to diabetes and improve diabetes detection for women with a history of GDM. Once diagnosed, efforts to promote lifestyle changes and increase metformin use may help delay or prevent diabetes and its complications.
